# Serum S100A6 Concentration Predicts Peritoneal Tumor Burden in Mice with Epithelial Ovarian Cancer and Is Associated with Advanced Stage in Patients

**DOI:** 10.1371/journal.pone.0007670

**Published:** 2009-10-30

**Authors:** Bih-Rong Wei, Shelley B. Hoover, Mark M. Ross, Weidong Zhou, Francesco Meani, Jennifer B. Edwards, Elizabeth I. Spehalski, John I. Risinger, W. Gregory Alvord, Octavio A. Quiñones, Claudio Belluco, Luca Martella, Elio Campagnutta, Antonella Ravaggi, Ren-Ming Dai, Paul K. Goldsmith, Kevin D. Woolard, Sergio Pecorelli, Lance A. Liotta, Emanuel F. Petricoin, R. Mark Simpson

**Affiliations:** 1 Molecular Pathology Unit, Laboratory of Cancer Biology and Genetics, Center for Cancer Research, National Cancer Institute, Bethesda, Maryland, United States of America; 2 Center for Applied Proteomics and Molecular Medicine, George Mason University, Manassas, Virginia, United States of America; 3 University of Brescia, Brescia, Italy; 4 Anderson Cancer Institute, Memorial Health University Medical Center, Inc., Savannah, Georgia, United States of America; 5 Data Management Services, Inc., National Cancer Institute, Frederick, Maryland, United States of America; 6 Centro di Riferimento Oncologico, IRCCS, National Cancer Institute, Aviano, Italy; 7 Antibody and Protein Purification Unit, Center for Cancer Research, National Cancer Institute, Bethesda, Maryland, United States of America; 8 Department of Haematology, Oncology, and Molecular Medicine, Istituto Superiore di Sanita, Rome, Italy; The University of Hong Kong, Hong Kong

## Abstract

**Background:**

Ovarian cancer is the 5th leading cause of cancer related deaths in women. Five-year survival rates for early stage disease are greater than 94%, however most women are diagnosed in advanced stage with 5 year survival less than 28%. Improved means for early detection and reliable patient monitoring are needed to increase survival.

**Methodology and Principal Findings:**

Applying mass spectrometry-based proteomics, we sought to elucidate an unanswered biomarker research question regarding ability to determine tumor burden detectable by an ovarian cancer biomarker protein emanating directly from the tumor cells. Since aggressive serous epithelial ovarian cancers account for most mortality, a xenograft model using human SKOV-3 serous ovarian cancer cells was established to model progression to disseminated carcinomatosis. Using a method for low molecular weight protein enrichment, followed by liquid chromatography and mass spectrometry analysis, a human-specific peptide sequence of S100A6 was identified in sera from mice with advanced-stage experimental ovarian carcinoma. S100A6 expression was documented in cancer xenografts as well as from ovarian cancer patient tissues. Longitudinal study revealed that serum S100A6 concentration is directly related to tumor burden predictions from an inverse regression calibration analysis of data obtained from a detergent-supplemented antigen capture immunoassay and whole-animal bioluminescent optical imaging. The result from the animal model was confirmed in human clinical material as S100A6 was found to be significantly elevated in the sera from women with advanced stage ovarian cancer compared to those with early stage disease.

**Conclusions:**

S100A6 is expressed in ovarian and other cancer tissues, but has not been documented previously in ovarian cancer disease sera. S100A6 is found in serum in concentrations that correlate with experimental tumor burden and with clinical disease stage. The data signify that S100A6 may prove useful in detecting and/or monitoring ovarian cancer, when used in concert with other biomarkers.

## Introduction

Ovarian cancer (OVCA) accounts for only 4% of cancer cases in women, yet it is the fifth leading cause of cancer death and the most lethal gynecological cancer in this population [1]. In 2008, there were an estimated 21,650 new cases and 15,520 deaths in the US [1]. Cisplatin, a platinum-based chemotherapeutic introduced in 1978, has become an essential part of an OVCA chemotherapy regimen and has greatly improved the outcome of early stage OVCA [2]; the 5-year survival rate for stage I patients is greater than 94% (http//:seer.cancer.gov/csr/1975_2006). Unfortunately, OVCA is rarely diagnosed at early stage when the disease is confined and often asymptomatic. Nearly 70% of OVCA cases are detected at disseminated stages, i.e. stages III and IV, during which the 5-year survival rate decreases to 30% or less.

An urgent OVCA research priority is the discovery and validation of biomarkers useful for diagnosing the most deadly types of OVCA, which often progress rapidly [3]. The only available FDA-approved non-invasive procedure for ovarian cancer diagnosis to date is the measurement of serum CA-125 levels. Even though 80% of patients with advanced OVCA have elevated serum CA-125, there is a high false positive rate associated with the CA-125 test [4]–[6]. Physical conditions such as pregnancy, pelvic inflammatory disease, benign cysts, uterine fibroids, or infection may also increase serum CA-125 levels [7], [8]. Other malignancies including pancreatic, lung, breast, gastric and colon cancers have also been shown to increase serum CA-125 [4], [8].

The emergence of mass spectrometry (MS) proteomics technology has brought new opportunities for discovering specific protein markers for early OVCA detection. Human serum stands as an attractive specimen for biomarker discovery using MS because sample acquisition is minimally invasive, and serum is the standard physiological fluid used for diagnostic purposes. However, the complexity and wide dynamic range of serum protein concentration make analysis of a total serum proteome challenging; serum protein concentrations vary >9 orders of magnitude and 99% of total serum protein mass is constituted by only approximately 22 protein species [9]. Such challenges associated with serum proteomics for biomarker discovery will not be easily overcome [8], [10]. Therefore, additional experimental approaches incorporating MS technology and serum sample processing should be examined in order to discover clinically relevant OVCA biomarkers. Indeed, methods such as depletion of abundant proteins using affinity columns and protein fractionation have been employed to increase the probability of uncovering tumor-derived protein species, which are often in low abundance [11]. An approach holding significant potential is analysis of the low molecular weight serum proteome/peptidome [12]. Low molecular weight (LMW) proteins and peptides often bind to high molecular weight serum proteins, thereby prolonging half-lives of the LMW fraction in circulation [13]–[15]. Thus, serum LMW proteome represents an attractive reservoir where tumor-derived low abundant proteins and peptides may be better preserved and potentially detected.

The development and use of OVCA animal models may serve as supplemental aids in identifying and confirming predictive serum biomarkers. The use of animal models has the prospect of minimizing some of the profound genetic and environmental variability often encountered in human proteome studies where unbiased control samples are often difficult to obtain [16]. Such studies using human cancer xenograft models have been published previously [17]–[20]. Transplantation of human cancer in immunodeficient mice is a useful model for the discovery of proteomic serum or plasma profiles that correlate with tumor burden. These models have the added benefit of providing means to determine if the biomarker of interest is derived directly from the cancer cell or is a product of the host response because one can examine the presence of tumor cell-derived human specific proteins. Target biomarkers identified by MS could then be further validated in such models, thereby increasing the probability of obtaining a pathologically relevant marker. Consequently, a human OVCA mouse model was established in an attempt to discern ovarian cancer-derived proteins in serum using MS-based discovery. This approach revealed the presence of S100A6, in addition to other human proteins, in sera from mice with OVCA. Expression of S100A6 was further examined in human cancer cell lines and tissue derived from OVCA patients. In addition, a system was sought to correlate quantity of serum S100A6 and tumor burden in the model. The latter aim endeavors to begin addressing important unanswered questions regarding a tumor size necessary to permit *de novo* detection of protein signatures of the mass in the blood. Lastly, we sought to validate the expression profile of S100A6 in human sera using a well-controlled clinical study set of women with early and advanced stage ovarian cancers.

## Results

**Figure 1:**
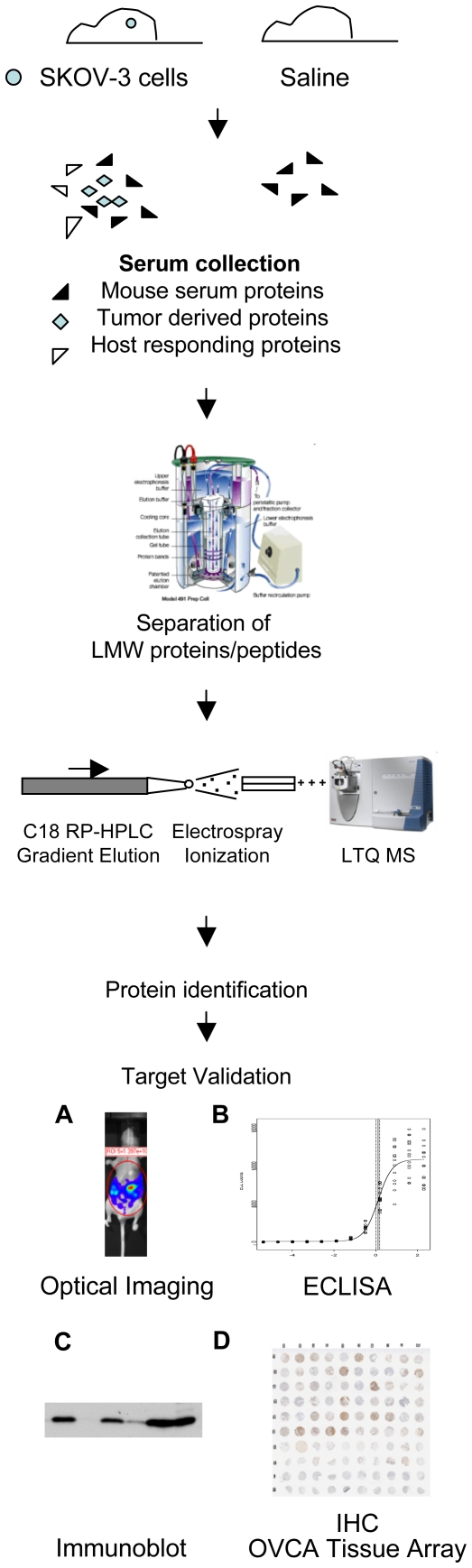
Experimental strategy to use MS discovery proteomics in low molecular weight (LMW) serum fraction from a mouse model of human ovarian cancer. To determine whether cancer-derived proteins have candidate biomarker potential, (A) a bioluminescent xenograft model, (B) ECLISA, (C) Western blot, and (D) OVCA tissue array immunohistochemistry (IHC) are utilized. This was followed up for S100A6 by analysis of human patient sera.

A general experimental approach for discovering LMW serum proteins with possible relevance for human OVCA in a mouse model using MS is depicted in Figure 1. Following intraperitoneal (i.p.) injection of mice with human serous SKOV-3 OVCA cells or saline control, blood was sampled at various time points while early and progressive carcinomatosis was being modeled. The LMW serum proteome was analyzed to identify proteins specific to, or more abundant in, cancer-bearing mice. The presence of tumor-derived S100A6 protein in serum was further studied as a candidate biomarker using antibody-based analyses. Tumor burden was correlated with the presence or level of serum protein S100A6 in the model, which thereby could be considered as a putative biomarker. The groundwork for further validating the clinical relevance of these findings for OVCA patients was laid by detecting more abundant S100A6 in sera from women with advanced stage disease, compared to early stage disease.

### Discovery of OVCA Associated Serum Proteins

**Figure 2:**
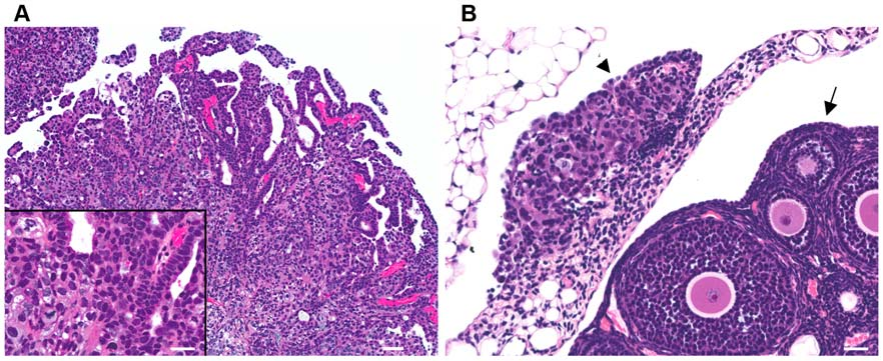
Human OVCA cell xenograft in mouse peritoneal cavity. (A) Representative OVCA exhibits predominantly solid tumor growth, with evidence of papillary projections and occasional minute cysts (bar = 25 µm). Inset, higher resolution photomicrograph depicting cuboidal to polygonal pleomorphic microcystic epithelium with anisokaryosis and atypical mitoses of ovarian cancer cell nuclei (bar = 50 µm). (B) OVCA tumor nodule implant on uterine infundibulo-ovarian ligament (arrowhead) adjacent to the ovary (arrow) (bar = 25 µm). Tumor implants occurred throughout the peritoneal cavity, peri-ovarian connective tissues, and invaded surrounding tissues such as intestines, liver and diaphragm. Hematoxylin and eosin (H&E) stained, paraffin-embedded tissue sections.

The experimental disease progressed from neoplastic cellular seeding of the peritoneum during engraftment to disseminated carcinomatosis, mimicking the progression from regional to distant advanced stage OVCA in women. All SKOV-3-inoculated mice exhibited multiple, variably-sized infiltrative tan, solid tumor nodules distributed throughout the peritoneum by 4 weeks post inoculation (p.i.). This was accompanied by neoplastic effusions in abdominal cavities of most SKOV-3-inoculated mice. Histologically, mesenteric masses were composed of a morphologically variable population of neoplastic ovarian epithelial cells. These cells occurred as expansile, papillary growths with trabeculae 2–3 cells thick, or as solid densely cellular masses supported by limited fibrous tissue (Figure 2A and B). All saline-inoculated control mice remained free of disease.

Index sera harvested from mice with late-stage carcinomatosis at 4 weeks following inoculation of 1×10^6^ SKOV-3 human OVCA cells were analyzed by MS and compared to control mouse sera (first of 3 study animal cohorts). The data were scrutinized to determine peptides/proteins with greater abundances in cancer vs. control samples (spectral count analysis). Overall the analyses yielded identification of about 400 peptides corresponding to approximately 300 proteins (combined human and mouse database search results). To determine proteins with a high likelihood of differential abundance, the results of the spectral count analysis of the human protein database search were filtered using Scaffold software to yield proteins with a minimum of 10 MS/MS spectra assigned (total of all replicate analyses) and a statistically significant difference in the number of assigned spectra (cancer>control) as measured by a t-test (p-value<0.05), or a minimum of a 100% difference in the spectral counts ((cancer counts - control counts)/(average cancer and control counts)×100%). These results are shown in Table 1.

**Table 1:** Differentially abundant proteins in serum from OVCA and control (C) mice selected based upon spectral counting.

Protein	Accession	MW	t-test	Spectra	Unique Human	Common Human/Mouse
	no.	(kDa)	p-value	(OVCA, C)	Peptide Sequences	Peptide Sequences
Fructose-bisphosphate aldolase C	P09972	39	2.0E-03	30, 0	GVVPLAGTDGETTTQGLDGLSER	
Keratin, type II cytoskeletal 1	P04264	66	2.0E-02	16, 0	YEELQITAGR	
Calcyclin (S100A6)	P06703	10	1.4E-04	29, 0	LMEDLDR	ELTIGSK; LQDAEIAR
L-lactate dehydrogenase A chain	P00338	37	4.0E-04	28, 8 *	DYNVTANSK; VTLTSEEAR	SADTLWGIQK
Fructose-bisphosphate aldolase A	P04075	39	2.0E-03	35, 0	AAQEEYVKR	ALQASALK
						GILAADESTGAIAK
Triosephosphate isomerase	P60174	27	9.0E-04	13, 0		VTNGAFTGEISPGMIK
Actin, cytoplasmic 1 (Beta-actin)	P60709	42	1.5E-02	18, 0		VAPEEHPVLLTEAPLNPK
Fibrinogen beta chain precursor	P02675	56	3.0E-01	12, 0		EDGGGWWYNR
Fibronectin precursor	P02751	63	6.0E-01	6, 1		VGDTYERPK
Coagulation factor X precursor	P00742	55	8.0E-01	9, 1		MLEVPYVDR
Apolipoprotein E precursor	P02649	36	5.0E-01	14, 2		LGPLVEQGR
Proteasome subunit alpha type 6	P60900	27	6.0E-02	18, 3		3 common sequences
Proteasome subunit alpha type 3	P25788	28	8.0E-02	17, 4		2 common sequences
Actin, aortic smooth muscle	P62736	42	2.0E-05	35, 8		3 common sequences

*One peptide sequence identified in controls is unconfirmed and differs from those identified in cancers.

Following identification of these candidate differentially abundant (cancer > control) human proteins, human peptide sequences identified by MS/MS spectra were then searched against a mouse database to examine if the peptide sequence was homologous between human and mouse and to verify the appropriateness of the sequence call (or if there was ambiguity in amino acid designation). Proteins were classified by having (1) human-specific peptides only, (2) peptides homologous to both human and mouse, and (3) both 1 and 2 (Table 1). Proteins identified by human-specific peptides are of potentially greater value since they most likely originated from the xenografted human cancer cells.

**Figure 3:**
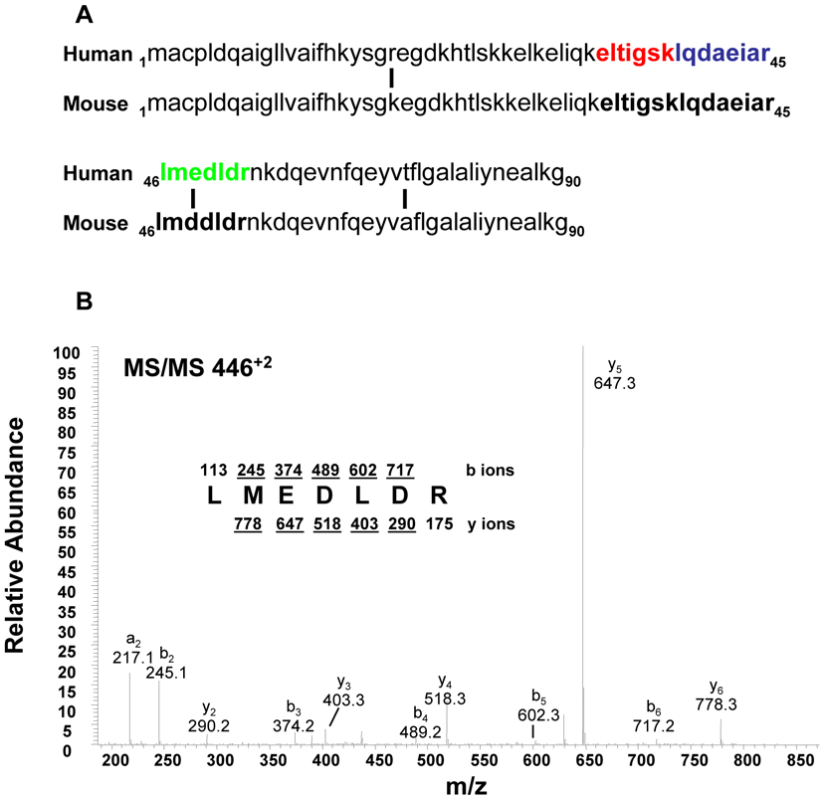
S100A6 protein sequence. (A) Aligned human and mouse S100A6 sequences depicting 3 amino acid differences between the two species (vertical lines). Three peptides were detected by LC-LTQ MS/MS from tumor-bearing mouse sera; sequences are shown in color font. Peptides in red and blue font are homologous for human and mouse, while the peptide sequence in green font is unique to human S100A6, due to one amino acid difference. (B) MS/MS spectrum of the human-specific S100A6 peptide, LMEDLDR. This sequence was not detected in saline-inoculated control mice sera.

S100A6 was selected for further study. In addition to meeting criteria for significance established for differentiating cancer associated serum proteins from control specimens (Table 1), S100A6 was regarded as a candidate for additional validation because of its increased expression in a variety of human cancers and its relatively small size (10.5 kDa). In the latter respect, S100A6 was attractive for its potential to be an intact protein yielded by the LMW separation strategy used to enrich for proteins with molecular weight less than ∼25 kDa. As shown in Table 1, three S100A6 peptides were identified; two sequences common to the human and mouse versions of this protein (red and blue fonts), and one peptide unique to the human version (green font) (Figure 3A). The MS/MS spectrum used to identify the unique human peptide, LMEDLDR, is shown in Figure 3B. The corresponding mouse sequence has an aspartic acid at the third residue position, instead of the glutamic acid in the human sequence. The presence of this human-specific peptide was subsequently confirmed in a second MS/MS analysis conducted on serum samples collected from the tumor-bearing mice in the third cohort of animals (see Materials and Methods).

### Expression of S100A6 in OVCA

**Figure 4:**
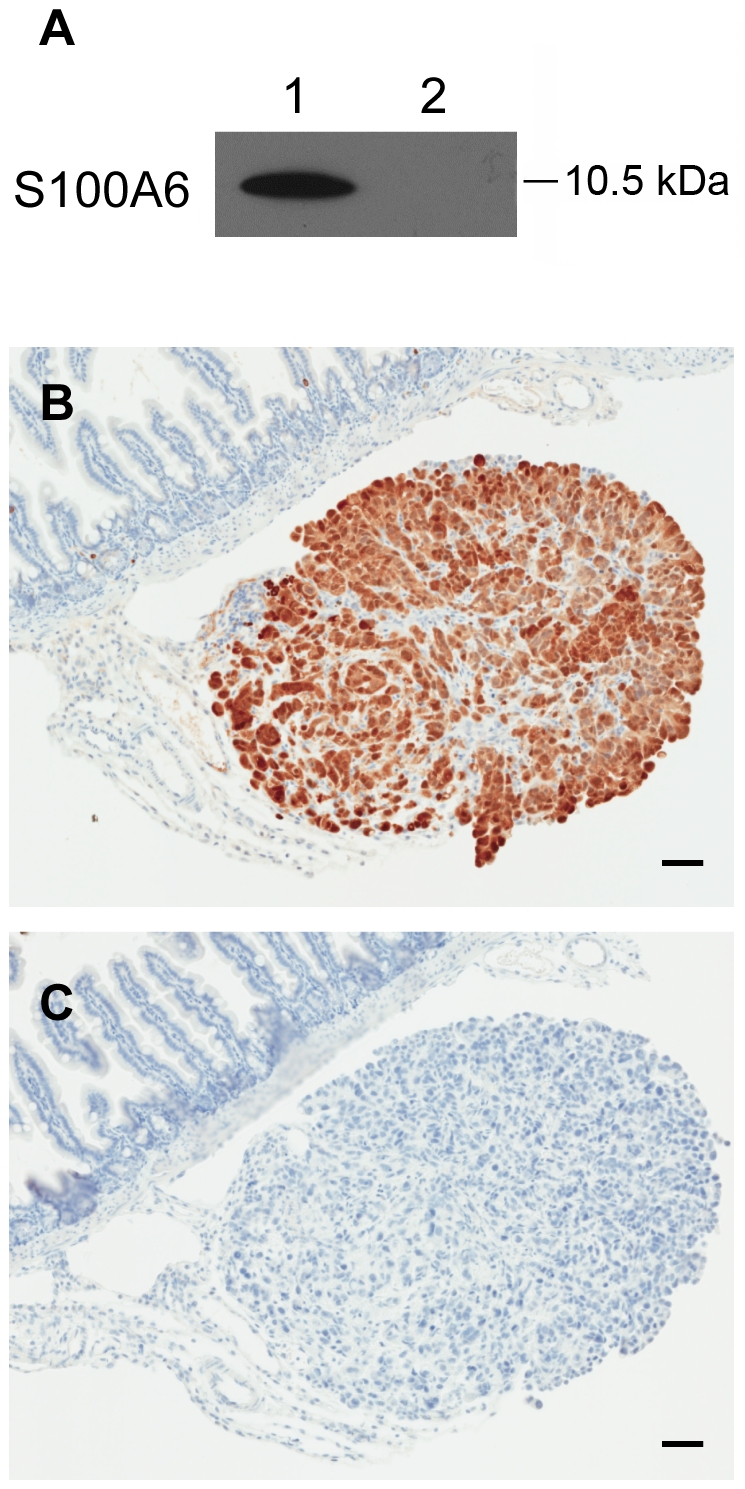
S100A6 protein expression in mouse serum and OVCA tissue. (A) Immunoblot analysis for the presence of S100A6 in pooled raw sera from cancer-bearing mice (1) and saline control mice (2). The sera were used in LMW protein fraction preparation for MS analysis in biomarker discovery. (B) OVCA xenografts express S100A6 as analyzed by IHC. Tumor xenograft implant on intestinal mesentery reveals S100A6 protein limited to tumor, with lack of immunolabeling in adjacent intestines and mesentery. (C) Serial section from tissue shown in B, used for IHC reaction control, reveals lack of immunoreactivity when primary S100A6 antibody is omitted from IHC. Immunoperoxidase, hematoxylin counterstain (Bars = 50 µm).

An anti-S100A6 immunoblot was carried out to confirm the presence of intact S100A6 in mouse sera used in the MS analyses. Using a custom anti-human S100A6 antibody, C1, a 10.5 kDa band corresponding to the molecular weight of S100A6 was detected in pooled sera collected 4 weeks p.i. from tumor-bearing mice used for initial MS analyses (Figure 4A). S100A6 was not detected in sera from matched-study saline-control mice. The C1 anti-S100A6 immunoblot was also performed on additional series of sera sampled from individual SKOV-3 cell-injected animals with experimental end stage disease, as well as sera from naïve control animals; the S100A6 10.5 kDa band was only observed in sera from animals with tumors, not in control mouse sera (data not shown). Evidence for S100A6 protein expression in OVCA xenografts and mouse abdominal organs was examined using immunohistochemistry (IHC). S100A6 immunolabeling was observed in tumor tissue but not in surrounding mesentery or abdominal organs (intestines, liver, kidney, urinary bladder, spleen, pancreas, uterus, and ovary) (Figure 4B and C).

**Figure 5:**
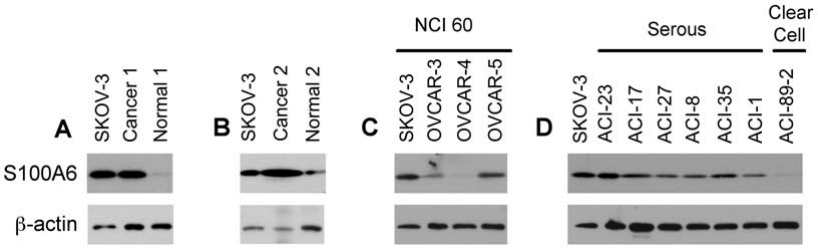
S100A6 expression in human OVCA cell lines and tissues by Western blot analysis. (A and B) Paired lysates of OVCA (cancer) and adjacent normal ovary tissue (normal) from 2 OVCA patients were blotted for S100A6 expression. SKOV-3 cell lysate was used for comparison. (C and D) S100A6 expression in OVCA cell lines from NCI60 (SKOV-3, OVCAR-3, 4 and 5) as well as cell lines derived from OVCA patients (see also Materials and Methods).

The potential clinical relevance of finding S100A6 in the sera of mice with SKOV-3 xenografts was further investigated by examining the expression of S100A6 in other OVCA human patient-derived specimens using Western blot. In paired cancerous and matched normal ovary lysates from 2 patients, S100A6 was elevated in carcinoma tissue compared to its matched non-neoplastic ovarian tissues (Figure 5A and B). Human OVCA-derived cell lines expressed various levels of S100A6 (Figure 5C and D). All OVCA cells tested, except OVCAR-4 and the cell line derived from clear cell ovarian carcinoma (ACI-89-2), expressed S100A6 above background level (normal ovary).

To further delineate differential S100A6 expression in human OVCA patient tissues, examination of malignant, borderline, benign, and noncancerous ovarian tissues was performed using IHC. Tissue sections lacking S100A6 immunoreactivity, similar to observations in the IHC negative reaction control, were considered negative. Immunoreactivity clearly above negative control was assigned as positive. Specimens with few numbers of weakly positive cells were considered equivocal. Among 140 malignant OVCA specimens, 116 (83%) were positive for S100A6 immunolabeling (Table 2). High S100A6 positive incidence (over 80%) was observed in all malignant types of OVCA with the exception of clear cell carcinoma, of which only 2 out of 4 specimens were S100A6 positive. Nearly 85% of benign adenomas showed positive S100A6 reactivity as well; the labeling intensity was comparable with that of malignant OVCA. No apparent relationship between labeling intensity and cancer stage or grade was observed. By contrast, normal ovarian tissues and non-neoplastic tissue adjacent to tumors exhibited limited or no S100A6 labeling. These results, demonstrating S100A6 expression in a variety of patient-derived epithelial ovarian cancer cell lines and tissues, establish that expression of S100A6 in OVCA is fairly common. In this context, finding human-specific S100A6 peptide sequence in serum from human OVCA-bearing mice indicates that S100A6 may have benefit as a candidate protein for monitoring OVCA. Furthermore, positive expression of S100A6 in benign and malignant OVCA, in contrast to its virtual absence on the surface of normal ovary, suggests S100A6 may be suitable as a marker in diagnostic imaging to help locate and corroborate ovarian proliferative changes.

**Table 2:** S100A6 immunohistochemical findings in epithelial OVCA and ovary tissues.

Ovary Pathology Diagnosis		No. of Patient Cases	Positive (%)	Negative (%)	Equivocal (%) *
**Malignant**					
Serous Adenocarcinoma		87	70 (80%)	14 (16%)	3 (4%)
Mucinous Adenocarcinoma		13	13 (100%)	0 (0%)	0 (0%)
Endometrioid Adenocarcinoma		20	16 (80%)	2 (10%)	2 (10%)
Transitional Cell Carcinoma		6	5 (83%)	1 (17%)	0 (0%)
Clear Cell Carcinoma		4	2 (50%)	2 (50%)	0 (0%)
Metastatic Serous Adenocarcinoma		10	10 (100%)	0 (0%)	0 (0%)
	**Total**	**140**	**116 (83%)**	**19 (13.5%)**	**5 (3.5%)**
**Borderline**					
Borderline Serous/Mucinous Cystadenoma		**5**	**4 (80%)**	**1 (20%)**	**0 (0%)**
**Benign**					
Benign - Adenomas		**13**	**11 (85%)**	**1 (7.5%)**	**1 (7.5%)**
**Normal**					
Non-Neoplastic Tissue Adjacent to Tumor		16	2 (13%) †	13 (81%)	1 (6%)
Morphologically Normal Ovary		13	0 (0%)	12 (92%)	1 (8%)
	**Total**	**29**	**2 (7%)**	**25 (86%)**	**2 (7%)**

*Equivocal  =  weak labeling or rare positive cells. † Reactive hyperplastic ovarian surface epithelium.

### Serum S100A6 Level Correlates With OVCA Tumor Burden

**Figure 6:**
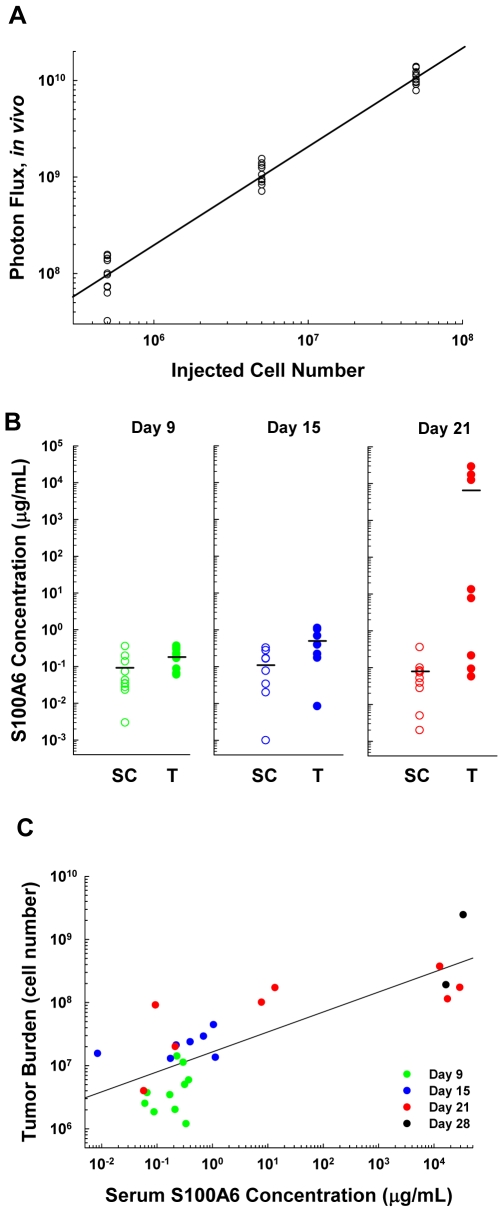
Detection of S100A6 in serum over time from mice with OVCA, in association with estimated peritoneal cavity tumor burden. (A) Photon flux measurements obtained from mice harboring defined numbers of bioluminescent SKOV-3-Luc cells within the peritoneal cavity. Regression analysis was performed on (log_10_) photon output vs. (log_10_) numbers of inoculated cells to produce a standard calibration curve using an inverse prediction equation (see Materials and Methods). This was used to predict tumor burden from photon flux measurements (see also figure 6C). (B) Comparisons of serum S100A6 in tumor-bearing (T, closed circles) and saline-inoculated control mice (SC, open circles). Serum S100A6 concentrations, tested as individual specimens from multiple mice by ECLISA (plotted as individual values with horizontal line at median value), were significantly different between T vs. SC at each of three times tested post inoculation, 9 days (p = 0.015), 15 days (p = 0.036), and 21 days (p = 0.0031) (Wilcoxon Rank Sum test). (C) Correlation between estimate of tumor burden in OVCA carcinomatosis and concentration of serum S100A6, as measured by ECLISA from tumor-bearing mice (r = 0.79, p<0.0001).

To determine whether the quantity of S100A6 in serum could be used as a correlate for estimating tumor burden, *in vivo* bioluminescent imaging and a modified ELISA assay (ECLISA) were applied. First, to provide estimates of tumor burden in cell numbers, 35 animals were injected with varying numbers of luciferase expressing SKOV-3 cells (SKOV-3-Luc) and *in vivo* bioluminescent photon signals were quantified (second cohort, Human Ovarian Cancer Animal Model, Materials and Methods). Regression analysis was performed on (log_10_) photon output vs. (log_10_) numbers of inoculated cells to produce a standard calibration curve (Figure 6A). An inverse prediction equation (see Materials and Methods) was derived from the regression analysis to obtain estimates of cell numbers, i.e., predicted tumor burden from the photon flux output of live animals.

To determine whether serum S100A6 levels escalated as tumor burden increased with time on study, mice were injected with SKOV-3-Luc or saline (third cohort, Human Ovarian Cancer Animal Model, Materials and Methods). On days 9, 15, 21 and 28 p.i., all SKOV-3-Luc-inoculated mice were optically imaged to obtain the photon output from growing cancers. Photon flux data were then converted to estimates of *in vivo* tumor cell burden. Ten animals each from tumor-bearing and control groups were bled and removed from study at each time point; serum S100A6 level was quantified by ECLISA for all study mice. Comparisons of serum S100A6 concentration from tumor-bearing mice and saline-injected controls revealed that, as early as 9 days p.i., there was significantly greater serum S100A6 concentration in the tumor-bearing mice, well before any identifiable tumor masses were present; the significant differential serum S100A6 level persisted through day 15 to day 21 p.i. (Figure 6B). Most day 28 p.i. serum specimens were used for MS/MS analysis, and the numbers of remaining serum samples were insufficient for this statistical comparison.

The correlation between tumor burden and the serum S100A6 level in specimens from these same SKOV-3-Luc-injected mice was sought next. When tumor burden (cell number) was plotted against serum S100A6 protein concentration in these animals, results indicated a highly significant correlation (r = 0.79, p<0.0001); serum S100A6 concentration was greater as the tumor burden increased over time for animals given SKOV-3-Luc (Figure 6C). The amount of S100A6 in saline-injected control mice remained near a basal level at all study time points, typically approximating the lower limit of S100A6 ECLISA detection (Figure 6B, SC). Escalating concentrations of S100A6 were also observed in sera collected serially at 1, 2, and 4 weeks p.i. from individual mice within the initial cohort of 20 mice given the parental SKOV-3 cell line (data not shown).

### Serum S100A6 Levels in Human OVCA Study Sets Are Linked To Disease Stage

**Figure 7:**
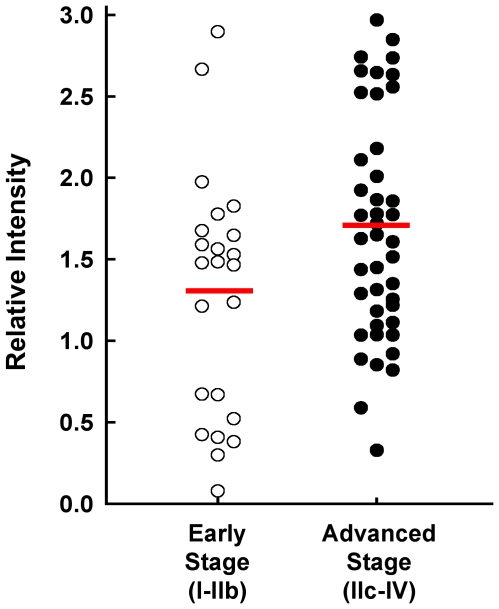
Reverse phase protein micorarray data obtained from the analysis of diagnostic sera from women with OVCA. Scatterplot displays relative intensity of serum S100A6 values for women with early stage (open circles, n = 23) and advanced stage (closed circles, n = 43) OVCA. The early stage disease group includes 2 tumors diagnosed as borderline OVCA. The means of the relative intensity values of the analyte concentrations shown (horizontal lines) are significantly different for the two groups (p = 0.031, two-sample t-test), and depict relatively greater mean S100A6 concentration in sera of advanced stage patients.

In order to determine if there was an association between elevated S100A6 protein and tumor burden in women with OVCA, we performed a pilot experiment using a well-controlled human clinical study set of 66 diagnostic serum samples that was available to us through the USA-ITALY Oncoproteomics Program. Sera had been obtained prior to therapy from women with early stage (I-IIb) and borderline OVCA (n = 23), and from women with advanced stage (IIc-IV) disease (n = 43). Immunoblots, with the C1 anti-S100A6, were carried out on a reverse phase protein microarray (RPMA) slide. Using this technique, levels of S100A6 were found to be statistically elevated in the sera of women with advanced stage disease compared to those with early stage tumors (p = 0.031), demonstrating an association between clinical features of greater tumor burden and increased levels of S100A6 in OVCA patients (Figure 7).

## Discussion

S100A6 was identified in the sera of mice with human OVCA using an MS/MS-based bottom-up proteomics strategy in an attempt to discover candidate biomarkers derived from tumor mass. The human tumor-origin of the S100A6 was established for the model, and the serum concentration of S100A6 correlated with the amount of experimental cancer present. Further clinical relevance was demonstrated through discovery of the potential for serum S100A6 to correlate with tumor burden using human clinical study sets.

During the discovery phase, a serum separation method to isolate the LMW proteome was employed to enrich proteins/peptides circulating in relatively low abundance, addressing the complexity of the whole serum proteome. The human SKOV-3 cell xenograft mouse model provided the opportunity to evaluate species differences in the effort to classify cancer-associated proteome as either tumor-derived (human) vs. host response (mouse). More in depth clinical validation studies are warranted to examine the potential for serum S100A6 to serve as a candidate biomarker for the evaluation of women with OVCA, particularly for monitoring recurrence. This premise is supported by the following evidence: (1) presence of a human-specific peptide sequence of S100A6 in sera, (2) detection of S100A6 protein expression in experimental tumors but not in surrounding non-neoplastic mouse tissues, (3) a positive and direct correlation between S100A6 serum concentration and tumor burden in the xenograft model, (4) demonstration of elevated S100A6 expression in tissues and cell lines from OVCA patients, but rarely in normal ovary, in this and other studies [21], [22], and finally, (5) significant elevation of the analyte in the sera of women with advanced stage OVCA vs. women with early stage disease. Limiting initial screening in mice to the SKOV-3 cell line in this case therefore did not preclude identifying a possible clinical surrogate biomarker for further validation. Additional human OVCA tissue-associated proteins were also reported in serum using this approach, and these can be tested further for candidate biomarker potential using the strategy applied here.

S100A6 was pursued for initial validation as a candidate serum biomarker due to its association with cancer progression [22], [23]. S100A6 is a small calcium binding protein that belongs to the S100 protein family. The approximately 25 members of the S100 family are characterized by 2 EF-hand structures and likely have calcium sensor function [24]. S100 proteins have been implicated in a wide range of physiological and pathological processes including cell proliferation, differentiation and intracellular signaling, although their exact functions remain to be defined further [24]–[27]. With respect to S100A6, its expression is normally found in fibroblasts and epithelial cells [28], and elevated S100A6 has been reported in a number of human cancer types including carcinomas of colon, pancreas, breast, ovary, lung and thyroid gland [29]–[35] .

There are conflicting reports about the role S100A6 plays in cancer. Vimalachandran et. al. [29] studied the expression pattern of S100A6 as it pertained to clinical outcome in pancreatic cancer and S100A6 expression was a negative prognostic factor. High, specifically nuclear, expression of S100A6 was associated with a poor prognosis. In addition, a stepwise increase of S100A6 during pancreatic carcinogenesis has been reported [36]. By contrast, elevated levels of S100A6 decreased the mobility of osteosarcoma cells and were associated with decreased clinically evident metastases [37]. Similarly, increased S100A6 expression was associated with a survival benefit for non-small cell lung cancers [35]; S100A6 was demonstrated in 2 lung cancer patients' sera, and in the pleural effusion of one. In the present study, we have extended the value of S100A6 to include it as an OVCA serum biomarker by demonstrating positive expression in human OVCA using immunohistochemistry, as well as an association between S100A6 serum level and tumor burden in both mice and human patients. The fact that S100A6 is elaborated by a number of human cancers, suggests that its use as a sole biomarker may encounter potential false positive incidences, akin to CA-125. Therefore, incorporating S100A6 into a panel of cancer biomarkers represents a logical approach to be pursued.

The low incidence of OVCA makes establishing and validating clinical biomarkers using human specimens difficult. Only 1 in 2500 post-menopausal women are estimated to be affected by OVCA annually [38], [39]. Because of this low incidence, population screening requires a large number of patients and therefore is very expensive for OVCA biomarker discovery and validation. For this reason the use of an animal model can be a cost efficient means to provide preclinical data to justify a large clinical validation study. Utilizing OVCA animal models for this purpose necessitates fidelity to both experimental aims and recapitulating cardinal features of human disease biology. The host of gene-targeted mutant mice developed for functional genomic studies has overshadowed application of human xenograft cancer models in some arenas recently [40]. Certain limitations of using mouse xenograft models for developing candidate biomarkers, such as engraftment of cultured cells, use of an immunocompromised host, and absence of cancer initiation from the ovary itself, must be acknowledged to permit a more precise focus on how data may be best applied to clinical disease. Nevertheless, the OVCA disease features produced in this study, recapitulating early neoplastic cell seeding of peritoneum and progression to advanced carcinomatosis, represents typical clinical case management situations. Indeed, over the last decade more than 80% of patients presented with metastatic disease within the pelvis or abdomen at the time of diagnosis [41]. Features of the experimental pathology faithfully recapitulate more advance stage disease biology (stages III and IV). Consequently, discovery of S100A6 as a surrogate for tumor burden in the model appears to have been predictive of the findings in women with advanced stage OVCA. Such a relationship further suggests that the model may be suitable for mimicking re-exacerbation of disease post surgical debulking, and for use in developing markers for patient monitoring. Concerns about limitations on discovery using xenograft models must be balanced in light of the absence of an ideally representative OVCA animal model, as well as the relevant clinical pathophysiology being investigated [40], [42].

In the present study, the human disease model permitted elucidation of the kinetics of S100A6 during OVCA disease progression prior to its clinical application. This was accomplished by developing and applying a novel antigen capture immunoassay, ECLISA, permitting in-solution analysis with a lower limit of detection in the range of 10–50 ng/mL of serum. By our analyses, the concentration of S100A6 in the range of approximately 100 ng/mL was considered to represent approximately 2–5 million OVCA cells in the peritoneal cavities of mice. Five million pelleted SKOV-3 cells have a mass of ∼20 mg; thus the assay would presumably enable detection of a minimal tumor cell load of about a 0.08% or less tumor to body weight ratio. It is worth noting that LMW proteins/peptides often associate with high molecular weight proteins in circulation [12]–[15], and therefore the antigenic epitopes of bound LMW proteins and peptides may not be readily available for antibody recognition. This issue was taken into consideration while developing the S100A6 ECLISA. It is important to emphasize that evidence for high molecular weight protein binding in the case of S100A6 was not substantiated conclusively, however antibody detection of S100A6 in solution did require addition of detergent (0.16% SDS) in raw serum in order to render the analyte available for antibody binding and to obtain optimal ECLISA signals. The RPMA employed used the same C1 anti-S100A6 that was used in the ECLISA, providing independent validation of S100A6 detection in serum. Other investigations comparing findings obtained from analyses of sera by both RPMA and ELISA-based assays indicate that they yield strongly correlative results [43], [44].

In conclusion, this study demonstrates the presence of S100A6 in OVCA cancer sera for the first time and further documents the potential use of S100A6 as an OVCA biomarker. Using a human xenograft model simulating disseminated growth of OVCA, serum S100A6 level, derived from the tumor tissue, directly correlated with increasing tumor burden and disease progression. In keeping with the observations that S100A6 correlates with tumor burden in the mouse model, and supporting further exploration of this biomarker for recurrence monitoring, we found that women with advanced stage OVCA had relatively increased levels of S100A6 in their blood compared to women with early stage disease. Additional studies exploring more deeply the true positive predictive value of S100A6 either alone, or in combination with CA-125 and other protein candidates resulting from this study, in a monitoring setting, are required and are ongoing. Aims will include a focus on creating what will likely be panels of tumor-derived serum proteins for use as markers in various OVCA disease constellations.

## Materials and Methods

### Human Ovarian Cancer Cells

SKOV-3 ovarian cancer cell line was purchased from American Type Culture Collection (ATCC) (Manassas, VA). SKOV-3 cells were cultured in McCoy's 5A medium (ATCC, Manassas, VA) supplemented with 10% fetal bovine serum and 100 units/mL penicillin G sodium, 100 µg/mL streptomycin sulfate, and 292 µg/mL L-glutamine (Invitrogen Life Technologies, Carlsbad, CA). Luciferase-expressing cells were generated by infecting SKOV-3 cells with pCLNCX-luciferase retrovirus (SKOV-3-Luc) (a gift from Dr. Steven Libutti, NCI, Bethesda, MD). Human OVCA cell lines tested *in vitro* included OVCAR-3, 4, and 5, from NCI-60 cell lines (http://dtp.nci.nih.gov/docs/misc/common_files/cell_list.html), and a series of 7 OVCA cell lines derived by Dr. Risinger at the Anderson Cancer Institute, Savannah, GA (JIR, unpublished data). Cells were cultured in DMEM/F12 medium (Invitrogen Life Technologies, Carlsbad, CA) with the same supplements described above.

### Human Ovarian Cancer Animal Model

Five to seven week-old nude mice (Ncr *nu/nu*) were obtained from NCI (Frederick, MD), maintained in accredited animal facilities and used as stipulated by the U.S. Public Health Service Policy on Humane Care and Use of Laboratory Animals, in accordance with institutional reviews (http://oacu.od.nih.gov). Three cohorts of animals were used in this study. The initial cohort was comprised of 40 mice that were individually identified, bled, and given intraperitoneal (i.p.) injection of 1×10^6^ SKOV-3 or equal volume of sterile saline (APP Pharmaceuticals, Schaumburg, IL). Serial blood samples were collected from retro-orbital sinuses at 1, 2 and 4 weeks p.i. Additional blood samples collected at study end by cardiac puncture at 4 weeks p.i. were pooled from both 6 principal and control mice and used in MS analysis for serum proteomics comparing cancer vs. non-cancer control. Sera were separated using Microtainer tubes (BS, Franklin Lakes, NJ), and stored at −80°C until analyzed.

A second cohort of mice was injected with SKOV-3-Luc cells in order to provide estimates of the number of cells in OVCA tumor burdens by determining the functional relationship between bioluminescent signal output and cell numbers. Animals were injected i.p., as in the previous experiment, using escalating numbers (5×10^5^, 5×10^6^, or 5×10^7^) of SKOV-3-Luc cells, 10–15 mice per cell quantity. Two hours after cell inoculation, animals received i.p. injection of 3 mg luciferin (Thermo-Fisher Scientific, Rockford, IL) and were imaged using Xenogen IVIS 100 optical imager according the manufacturer's guide (Caliper Life Sciences, Hopkinton, MA). A standard calibration curve of known cell numbers inoculated vs. photon flux measurements was developed such that cell numbers, along with their confidence intervals, could be predicted by inverse regression calibration (see Serum S100A6 Quantification and Data Analysis below).

A third cohort of mice was inoculated with SKOV-3-Luc cells to provide a timed longitudinal study to examine the kinetics of serum S100A6 protein, identified by MS analysis of sera from the first cohort of mice, in its relationship to OVCA tumor burden. A cohort of 80 animals received 1×10^6^ SKOV-3-Luc cells (40 mice, tumor group) or saline (40 mice, control group) on day 0. On days 9, 15, 21 and 28 p.i., all live animals inoculated with cancer cells were optically imaged to obtain the bioluminescent signals from engrafted SKOV-3-Luc (Caliper Life Sciences, Hopkinton, MA). Ten mice in each group (cancer bearing and saline vehicle-inoculated control groups) were bled by cardiac puncture at each time point and removed from study. Sera from most of the mice with late-stage disease collected at 28 day p.i. were pooled for use in second MS analysis, intended to confirm the initial S100A6 results. Tumors (or mesenteric tissue from early time point and control mice) were excised at necropsy, immersed in formalin fixative and subsequently paraffin embedded routinely for histological examination and IHC.

### Mass Spectrometry

LMW serum proteins were prepared and analyzed as described [45]. In brief, pooled serum samples were prepared from tumor and control groups by combining equal volumes of serum from each mouse within the same group followed by thorough mixing. One hundred microliters of the pooled serum samples were mixed with 2x SDS-PAGE loading buffer (Invitrogen Life Technologies, Carlsbad, CA), boiled for 10 minutes and loaded onto an electrophoresis apparatus, Prep Cell (BioRad Laboratories, CA), packed with 10% acrylamide gel. Continuous flow denaturing electrophoresis was accomplished under a constant voltage of 250V. Immediately after the bromophenol blue dye front migrated out of the gel, LMW peptides and proteins were eluted in Tris-Glycine running buffer (0.3% Tris, pH 7.4, 1.44% glycine and 0.1% SDS) at a flow rate of 500 µL/minute for 5 minutes per fraction. Ten microliters of each fraction were loaded onto an SDS-PAGE gel to examine the molecular weight range of protein/peptide components in each fraction collected. Fractions containing proteins/peptides with molecular weights less than ∼25 kDa were combined and concentrated using a Centricon filter (Millipore, Billerica, MA) to a final volume of 300 µL. SDS was removed from the LMW protein samples by tricholoroacetic acid (TCA) precipitation, in which the concentrated proteins/peptides were incubated with an equal volume of 10% TCA (w/v) on ice for 1 hour and the mixture then was centrifuged at 15,000x g for 30 minutes at 4°C. The pellet containing the precipitated proteins/peptides was washed in cold acetone and dissolved in 8 M urea.

The SDS-free LMW fractions were analyzed using a traditional bottom-up MS proteomics method. The LMW peptides/proteins first were reduced and alkylated by reaction with 20 mM dithiothreitol followed by 100 mM iodoacetamide. The samples then were digested with trypsin (Promega, Madison, WI) at 37°C overnight in 50 mM ammonium bicarbonate in the presence of 1 M urea, and the resulting tryptic peptides were desalted using a μC_18_ Zip-Tip (Millipore, Billerica, MA). The peptide samples were analyzed by liquid chromatography (LC)-ESI (electrospray ionization)-MS/MS using an LTQ linear ion trap mass spectrometer (Thermo Scientific, San Jose, CA). An aliquot of each sample was loaded onto a home-made reverse-phase C_18_ column that consisted of a 10 cm long, 100 µm inner diameter piece of fused silica packed with 5 µm 200 Å pore size C_18_ resin (Michrom Bioresources, Auburn, CA) with an integrated laser-pulled tip. After loading the sample, the column was washed for 5 minutes with LC solvent A (0.1% formic acid) and then the peptides were eluted with a flow rate of 250 nL/minute using a linear gradient from 0% solvent B (0.1% formic acid, 80% acetonitrile) at 5 minutes to 50% B in 30 minutes and then to 100% B in an additional 5 minutes. The LTQ was operated in a data-dependent mode in which each full MS scan was followed by five MS/MS scans whereby the five more abundant ions in the MS scan were selected dynamically, fragmented by collision-induced dissociation and the fragment ions detected. Multiple replicate analyses of serum pool aliquots were performed.

The mass spectral data were searched against the Swiss-Prot databases of human and mouse proteins using the SEQUEST algorithm as part of the Bioworks software (Thermo Scientific, San Jose, CA) with a full tryptic cleavage requirement and a static cysteine alkylation modification. High confidence peptide identifications were obtained by filtering the search results based on the following criteria: minimum cross-correlation score of 1.8 (+1 ion), 2.2 (+2) and 3.5 (+3); minimum delta C_n_ of 0.1 and maximum probability of a random match of 0.01. These match criteria yielded false discovery rates of 1–5%. In addition, all matches corresponding to peptides that were deemed to potentially be differentially abundant in cancer vs. control samples were evaluated by manual inspection of the data. Comparative analysis based on spectral counting was accomplished using Scaffold (Proteome Software, Inc., Portland, OR).

### Cell Lysate Preparation and Western Blot Analysis

Paired OVCA and matched normal ovary tissue protein lysates, from two patients, were obtained from Abcam (Cambridge, MA). Cultured cells were lysed in RIPA buffer (Cell Signaling Technology, Danvers, MA) for 20 minutes at 4°C. Cell lysates were centrifuged at 13,000× g for 5 minutes to remove cellular debris. One microliter of mouse serum or 2.5 µg of clarified cell lysates were separated on 4–20% Tris-Glycine gradient gels (Invitrogen Life Technologies, Carlsbad, CA) and transferred to Immun-Blot PVDF membranes (Bio-Rad Laboratories, Hercules, CA). The membranes were blocked in 3% bovine serum albumin, blotted with primary antibodies followed by peroxidase-conjugated secondary antibodies (Jackson ImmunoResearch Laboratories, Inc. West Grove, PA). The antibody reactivity was detected using electrochemiluminescent (ECL) Western Blotting Substrate (Thermo Fisher Scientific, Rockford, IL). Rabbit anti-human S100A6, C1 (see S100A6 Quantification section) and mouse anti-human β-actin (Sigma Aldrich, St. Louis, MO) were used as primary antibodies at a working concentration of 10 ng/mL and 0.1 µg/mL, respectively.

### Immunohistochemistry

Immunohistochemistry (IHC) was performed as previously described [46], [47] with minor modifications. Briefly, 6 µm-thick tissue sections or high density ovarian tissue core microarray slides (OV1001 and OV2082, US Biomax, Rockville, MD) were deparaffinized and rehydrated. Antigen retrieval was performed by immersing tissues in target antigen retrieval buffer (TAR®, Dako, Carpinteria, CA) in a steamer (Black and Decker, Hunt Valley, MD) for 15 minutes, followed by 15 minutes cooling on the bench top. Primary antibody, mouse anti-human S100A6 antibody (Sigma-Aldrich, St. Louis, MO) at 1∶700 dilution, and biotinylated goat anti-mouse IgG secondary antibody (Dako, Carpinteria, CA) were used. Reactions were developed using a peroxidase-based streptavidin detection method (Vector Labs, Burlingame, CA) and 3,3′-diaminobenzidine tetrahydrochloride (DAB) (Invitrogen, Carlsbad, CA) as chromogen substrate. Negative reaction control included substitution of antibody diluent for the respective primary antibodies on tissue sections and array slides.

### Serum S100A6 Quantification by ECLISA and Data Analysis

Antigen-capture sandwich immunoassay employing electrochemiluminescence technology (ECLISA) (Meso Scale Discovery (MSD), Gaithersburg, MD) was developed to quantify serum S100A6 levels. Two custom rabbit anti-human S100A6 polyclonal antibodies were generated for this study. The capturing antibody, C1, was generated against amino acids 42–90 of human S100A6 (Primm Biotech, Inc. Cambridge, MA), while a detecting antibody, 9249B, was raised against a peptide corresponding to amino acids 82–90 of human S100A6 (Rockland, Gilbertsville, PA). Microtiter plates (MULTI-ARRAY, MSD, Gaithersburg, MD) were coated with 4 µg/mL capturing antibody (C1) and blocked with 1% casein. Test sera diluted in 0.16% SDS in phosphate buffered saline (PBS, pH 7.4) were added to the coated plates and incubated for 1 hour at room temperature. An MSD-TAG labeled secondary anti-S100A6 antibody (9249B), prepared according to manufacturer instructions (MSD, Gaithersburg, MD), was used to detect captured S100A6. A 620 nm electroluminescent signal was generated and detected using Sector Imager 2400 (MSD, Gaithersburg, MD).

A human recombinant S100A6 protein was used in establishing a standard curve to quantify serum S100A6 using the ECLISA. Four-fold serial dilutions of recombinant S100A6 protein starting from 20 µg/mL to 3.05×10^−4^ µg/mL were included (in triplicate) in each analysis performed. Additional assay control included analysis of the same known positive and negative serum samples with each run. ECLISA signals were plotted vs. the known concentrations and fit with a four-parameter nonlinear logistic regression model to obtain a standard curve of S100A6. The standard curve run for each assay was then used to back-calculate the S100A6 concentrations in serum samples, within that assay, from the ECLISA signals. During preliminary development, the four-parameter model was used to assess inter-run immunoassay precision of five standard curves generated on different dates. The five standard curves were found to be statistically equivalent with respect to their lower asymptote, ‘slope’ and ‘50% maximal response’ parameters.

Data in this study were analyzed using parametric and nonparametric statistical methods, correlation and regression techniques, and nonlinear hierarchical mixed-effects regression models [48]–[53] using S-Plus, R, SAS and MSD (MSD, Gaithersburg, MD) software [53]–[55]. Regression, inverse regression and calibration analyses were employed to determine whether S100A6 serum concentrations could be used as a correlate for tumor burden. Specifically, linear regression analysis was performed on (log_10_) photon flux output (bioluminescent signal) vs. (log_10_) numbers of inoculated cells to produce a standard calibration curve with intercept and slope parameter estimates 2.27 and 1.01, respectively. An inverse prediction equation: log_10_ (cell number) = (log_10_ (photon flux output)−2.27)/1.01 was derived from the regression analysis to obtain estimates of predicted cell numbers, along with their 95% confidence (fiducial) limits, to predict tumor burden from the photon flux output of live animals. Care was taken in the modeling process to satisfy homogeneity and homoscedasticity requirements in the regression/calibration analyses. ECLISA signals from serial dilutions of recombinant S100A6 protein were analyzed using the four-parameter nonlinear regression model, y = b_2_ + ((b_1_−b_2_ /(1 + ( x / b_3_ ) ^ b_4_)), in which b1, b2, b3, and b4 represent, respectively, lower and upper asymptotes, half-maximal concentration, and ‘slope’ factors [49]. Nonlinear regression model fits and back-calculations of S100A6 serum concentrations were obtained from software developed for this study using S-Plus and R [54], [55]. For the purpose of assigning S100A6 concentration values to 5 serum specimens with ECLISA signal responses above the calibration standard curve plateau, a working ECLISA signal value<0.01% below the upper asymptote was entered into the back-calculation computer routine to provide conservative estimates of serum S100A6 levels for these cases. Qualitatively, the relative concentration of these was verified by Western blot.

Comparisons of tumor vs. saline control treatment conditions were carried out using parametric (t-tests, Welch's modified t-tests for unequal variances) and nonparametric (Wilcoxon Rank Sum) statistical tests. Assumptions regarding homogeneity of variance were routinely examined and appropriate tests applied. For simplicity, probability values from the Wilcoxon Rank Sum test are reported for comparisons of tumor vs. saline control treatment conditions at days 9, 15 and 21, respectively for the mice in the 3^rd^ cohort. All statistical tests were two-sided; probability values less than 0.05 were considered significant.

### Human Sera Study Set

The study set involved 66 serum specimens collected with institutional approval at two Italian medical centres (Department of Obstetrics and Gynaecology, University of Brescia, and Centro di Riferimento Oncologico (CRO), Aviano). Samples were obtained from fasting patients either the day prior to, or the day of, surgical intervention, before induction of anaesthesia for treatment. Participation of patients and the collection of samples for the study were approved by the respective Institutional Review Boards, and written informed consent was obtained from all patients enrolled. Twenty-three (34.8%) specimens were classified as early stage (I-IIb) (median patient age, 53 (range 24–68)), and 43 specimens (65.2%) were advanced stage (IIc-IV) OVCA (median patient age, 60 (range 35–89)). Histologically, there were 20 serous, 5 endometrioid, 6 mucinous, 5 clear cell, and 13 undifferentiated OVCA. Two cases were classified as borderline tumors (one serous, one mucinous). There was 1 germ cell OVCA and 14 cancers were not otherwise specified. Blood samples were collected in 7.5 mL S-Monovette vials (Sarstedt, Verona, Italy) containing clot-activator and were maintained at room temperature for up to 30 to 90 minutes, after which all samples were centrifuged at 4000 rpm for 20 minutes. Serum was stored at −80°C in aliquots of 500 microliters.

### Immunoblotting of Reverse Phase Protein Microarray and Data Analysis

Human serum S100A6 levels were determined by reverse phase protein microarray (RPMA) technology. Serum handling, preparation, and analysis of arrays have been described previously [56], including successful application of the technology to low abundance analyte measurement in human sera [43], [44]. Briefly, sera (approximately 60 nL) had been arrayed in triplicate through multiple depositions as 350 µm diameter spots onto Nitrocellulose FAST slides (Whatman, Florham Park, NJ), in such a fashion to obtain even interposition of cases and controls throughout the slide, using a 2470 Arrayer (Aushon BioSystems, Burlington, MA). Slides were stored at −20°C with a desiccant (W.A. Hammond Co. Wenia, OH) until assayed. Controls included recombinant human S100A6 protein (diluted 1∶40 in extraction buffer) spiked into normal mouse serum (S100A6-negative by WB and ECLISA), and arrayed as calibration standards in a ten points dilution curve (1∶2) with starting concentration of 4.16 ng/mL. Three serum samples from mice, two with OVCA xenografts and one normal mouse, were included in the slide as additional positive and negative controls. Appropriate results were previously obtained for these control mouse samples using Western blot and ECLISA.

RPMA slides were blocked in I-Block (Applied Biosystems, Foster City, CA) and incubated with C1 anti-S100A6 at 73 ng/mL, followed by secondary antibody (BA-1000, Vector Labs, Burlingame, CA) at 1∶200,000 dilution. All washes were preformed with Tris-buffered saline (0.05 M Tris-HCl, 0.15 M NaCl, pH 7.6) with 0.1% Tween 20. Staining was performed by means of an automated slide stainer (DAKO, Carpinteria, CA) and signal was generated as previously described [56]. A negative slide probed with secondary antibody only was prepared to determine the level of background signal due to nonspecific binding of secondary antibody. A Sypro Ruby (Molecular Probes, Eugene OR) staining was carried out subsequently to determine the total protein quantity for normalizing the scanned optical staining intensity values of the primary antibody. Slides were scanned on a flatbed optical scanner using greyscale mode and 16-bit images/600dpi. ImageQuant analysis software (www.imsupport.com) was used to determine spot intensities. All spot intensity values were subjected to local background subtraction, secondary antibody alone background subtraction, and normalization relative to total protein intensities. These steps were taken to ensure that changes in levels of protein expression were not simply due to differences in overall protein volume or spotting variances. Final relative intensity values obtained were computed as means from the normalized background-corrected triplicate values for each sample. Early stage and advanced stage patients data were then compared by means of a two-sample t-test after testing for homogeneity of variance and normality.
